# An organoid model of colorectal circulating tumor cells with stem cell features, hybrid EMT state and distinctive therapy response profile

**DOI:** 10.1186/s13046-022-02263-y

**Published:** 2022-03-08

**Authors:** Maria Laura De Angelis, Federica Francescangeli, Chiara Nicolazzo, Michele Signore, Alessandro Giuliani, Lidia Colace, Alessandra Boe, Valentina Magri, Marta Baiocchi, Antonio Ciardi, Francesco Scarola, Massimo Spada, Filippo La Torre, Paola Gazzaniga, Mauro Biffoni, Ruggero De Maria, Ann Zeuner

**Affiliations:** 1 Department of Oncology and Molecular Medicine, Istituto Superiore di Sanità, Viale Regina Elena 299, 00161 Rome, Italy; 2 Department of Molecular Medicine, Liquid Biopsy Unit, Sapienza University, Viale Regina Elena 324, 00161 Rome, Italy; 3 RPPA Unit, Proteomics Area, Core Facilities, Istituto Superiore di Sanità, Viale Regina Elena 299, 00161 Rome, Italy; 4 Environment and Health Department, Istituto Superiore di Sanità, Viale Regina Elena 299, 00161 Rome, Italy; 5 Department of Surgical Sciences, Policlinico Umberto I/Sapienza University of Rome, Viale del Policlinico 155, 00161 Rome, Italy; 6 Core Facilities, Istituto Superiore di Sanità, Viale Regina Elena 299, 00161 Rome, Italy; 7 Department of Radiological, Oncological and Pathological Sciences, Sapienza University, Viale del Policlinico 155, 00161 Rome, Italy; 8 Department of Surgery “Pietro Valdoni”, Policlinico Umberto I/Sapienza University, Viale del Policlinico 155, 00161 Rome, Italy; 9 Center of Animal Research and Welfare, Istituto Superiore di Sanità, Viale Regina Elena 299, 00161 Rome, Italy; 10 Surgical Sciences and Emergency Department, Policlinico Umberto I/Sapienza University of Rome, Viale del Policlinico 155, 00161 Rome, Italy; 11 Dipartimento di Medicina e Chirurgia Traslazionale, Università Cattolica del Sacro Cuore, Largo Francesco Vito 1, 00168 Rome, Italy; 12 Fondazione Policlinico Universitario A. Gemelli IRCCS, Largo Agostino Gemelli 8, 00168 Rome, Italy

**Keywords:** Circulating tumor cells, Colorectal cancer, Organoids, Metastasis, Cancer stem cells

## Abstract

**Background:**

Circulating tumor cells (CTCs) are responsible for the metastatic dissemination of colorectal cancer (CRC) to the liver, lungs and lymph nodes. CTCs rarity and heterogeneity strongly limit the elucidation of their biological features, as well as preclinical drug sensitivity studies aimed at metastasis prevention.

**Methods:**

We generated organoids from CTCs isolated from an orthotopic CRC xenograft model. CTCs-derived organoids (CTCDOs) were characterized through proteome profiling, immunohistochemistry, immunofluorescence, flow cytometry, tumor-forming capacity and drug screening assays. The expression of intra- and extracellular markers found in CTCDOs was validated on CTCs isolated from the peripheral blood of CRC patients.

**Results:**

CTCDOs exhibited a hybrid epithelial-mesenchymal transition (EMT) state and an increased expression of stemness-associated markers including the two homeobox transcription factors Goosecoid and Pancreatic Duodenal Homeobox Gene-1 (PDX1), which were also detected in CTCs from CRC patients. Functionally, CTCDOs showed a higher migratory/invasive ability and a different response to pathway-targeted drugs as compared to xenograft-derived organoids (XDOs). Specifically, CTCDOs were more sensitive than XDOs to drugs affecting the Survivin pathway, which decreased the levels of Survivin and X-Linked Inhibitor of Apoptosis Protein (XIAP) inducing CTCDOs death.

**Conclusions:**

These results indicate that CTCDOs recapitulate several features of colorectal CTCs and may be used to investigate the features of metastatic CRC cells, to identify new prognostic biomarkers and to devise new potential strategies for metastasis prevention.

**Supplementary Information:**

The online version contains supplementary material available at 10.1186/s13046-022-02263-y.

## Background

Colorectal cancer (CRC) is the second leading cause of cancer death worldwide [1]. While early-stage CRC is associated with high survival rates, advanced disease remains usually incurable despite the improvement of therapeutic protocols [2]. Therefore, new tools are urgently needed for the investigation, prevention and treatment of metastatic disease. Circulating tumor cells (CTCs) contain the physical elements responsible for tumor dissemination and metastasis. CTCs analysis through liquid biopsies provides not only a full repertoire of tumor biological material (including proteins, lipids, sugars and nucleic acids) but also a sample of living tumor cells endowed with metastatic ability [3]. Thus, CTCs characterization offers the opportunity to gain mechanistic insights into the metastatic process and to exploit specific CTCs features for metastasis prevention and treatment [4, 5]. Despite the great potential of CTCs for cancer diagnosis, prognosis and therapy, the technical challenges associated to their isolation and enrichment hampered for long time a successful clinical use. Several experimental models have been developed to allow the expansion of CTCs from multiple tumors including lung, breast, esophageal, bladder, gastric, pancreatic, colorectal and prostate cancer, hepatocellular carcinoma and pleural mesothelioma [6–20]. Among these, 3D models such as spheroids and organoids provide an improved reconstruction of the tumor context [21–23], which can be further improved with the use of coculture systems [24, 25]. Furthermore, in vivo CTC-derived xenografts are currently under scrutiny as suitable models to investigate the metastatic process [8, 26, 27]. The development of CTCs-based preclinical models is tightly linked to overcoming the difficulties related to CTCs rareness. In fact, cultures derived from patients’ CTCs usually take many months to grow (which is incompatible with their use for treatment decision) and may be scarcely representative of tumor complexity [28]. Nonetheless, CTCs represent a unique source of patients’ material and a valuable in vitro proxy platform for anti-cancer drug testing. We have established an experimental model of CTCs-derived organoids (CTCDOs) obtained from circulating cancer cells spontaneously generated in an orthotopic CRC xenograft model derived from one CRC patient. CTCDOs recapitulated several features of CRC CTCs including a hybrid epithelial-mesenchymal transition (EMT) state and elevated expression of stemness-associated proteins. Moreover, CTCDOs displayed a distinctive pattern of drug sensitivity that may be helpful for the identification of anti-metastatic strategies. While our results will need confirmation on a larger number of patients, this study provides a proof of principle for the generation of organoid cultures with CTCs features starting from a primary tumor sample. Further validations of such experimental model may lead to new approaches for the identification of prognostic biomarkers, therapeutic targets and personalized treatment strategies.

## Methods

### Generation and validation of patient-derived organoids (PDOs), xenograft-derived organoids (XDOs) and circulating tumor cell-derived organoids (CTCDOs)

CRC was obtained from a 69 years old male patient with a G2 stage IVA left colon tumor upon informed consent and approval by the Policlinico Umberto I Ethical Committee (RIF.CE: 4107 17/10/2016). For CTCs isolation, mice blood was centrifuged using Lympholyte Cell separation media (#CL5020, Cedarlane Laboratories, Canada) and CTCs were selected from the mononuclear cell layer by depleting hematopoietic cells with ferromagnetic anti-mCD45 coated microbeads (#130-052-301, Miltenyi Biotec, Germany) as detailed in Additional file 1: Supplementary Methods. Organoid cultures were generated from either single cell suspensions from patient’s tissue, from subcutaneous xenografts or from CTCs isolated from mouse blood, by the method described in [29]. Shortly, cells were resuspended in Growth Factor Reduced Matrigel® (Corning). Matrigel® was overlaid with 500 μL of colon cancer culture medium supplemented with 20 ng/mL recombinant human epidermal growth factor (EGF), 10 ng/ml human basic fibroblast growth factor (bFGF) (both from Peprotech), 10 nM Gastrin, 10 μM Y-27632, 10 μM SB202190 (Sigma-Aldrich) and 500 nM A83-01 (Tocris Bioscience, UK).

### Antibodies and reagents

All antibodies are reported in Additional file 1: Supplementary Methods.

### Animal procedures

Animal procedures were performed according to the Italian national animal experimentation guidelines (D.L.116/92) upon approval of the experimental protocol by the Italian Ministry of Health’s Animal Experimentation Committee (DM n. 292/2015 PR 23/4/2015). 6-week-old female NOD.Cg-Prkdc^scid^ Il2rg^tm1Wjl^/SzJ (NSG) mice were used for all experiments. For orthotopic xenografting, 10^5^ cells obtained from dissociated PDOs and transduced with a luciferase (LUC)-expressing lentiviral vector were injected in the colon wall during open laparotomy and tumor formation was monitored with an IVIS imaging system (Perkin Elmer). For CTCs isolation, 1 mL of whole blood was drawn via transthoracic cardiac puncture. For subcutaneous xenografts generation, 5 × 10^4^ dissociated cells obtained from PDOs or CTCDOs resuspended in PBS/Matrigel were injected in the flank of NSG mice. Tumor volumes were calculated with the formula: π/6 x d2 x D, where d and D represent shorter and longer tumor measurements. For stem cell quantitation in xenografts, tumors were pooled and dissociated into single cells that were injected into secondary mice at serial doses ranging from 10 to 10.^3^ Five animals were used for each dilution point, and mice were recorded as negative when no graft appeared 24 weeks after inoculation.

### CTCs detection in mouse peripheral blood

CTCs were identified with the CellSearch® technology (Menarini Silicon Biosystems) as described in Additional file 1: Supplementary Methods.

### CTCs isolation from CRC patients

Peripheral blood was obtained from patients with metastatic CRC (Additional file 2: Table S1 and Additional file 1: Supplementary Methods) and CTCs were isolated with the Screen Cell Size-Isolation Device (ScreenCell®, France) according to the manufacturer’s instructions. All participants provided informed consent to the study.

### Immunohistochemistry, flow cytometry, immunofluorescence, clonogenicity assay and single cell cloning, invasion/migration assay and immunoblotting

Please refer to Additional file 1: Supplementary Methods.

### Drug screening

Anti-cancer compounds and low toxicity compounds listed in Additional file 3: Table S2 were purchased from Selleck Chemicals. Dissociated organoid cells were plated in Medium/Matrigel at 3500 cells/well in triplicate 72 h before drug treatment. Organoids were treated for 6 days in a humidified atmosphere at 37 °C, 5% CO_2_ and drug-containing medium was replaced every 72 h. Cell viability was determined with CellTiterGlo 3D viability assay (Promega). Further details are provided in Supplementary Methods, Additional file 1.

### Proteome profiler arrays

The expression of stem cell- cancer- and stress-related proteins was evaluated with Proteome Profiler Human Pluripotent Stem Cell Array Kit (#ARY010), Proteome Profiler Human XL Oncology Array (#ARY026) and Proteome Profiler Human Cell Stress Array Kit (#ARY018) all from R&D Systems. Protein list is available in Additional file 4: Table S3. Further details are provided in Supplementary Methods, Additional file 1.

### Statistical analysis

Statistical analyses were performed using GraphPad Prism version 4.0 for Windows (GraphPad Software) with unpaired Student’s t test. Results are presented as the mean ± SD or mean ± SEM where appropriate. Statistical significance is expressed as *, *P* < 0.05, **, *P* < 0.01 and ***, *P* < 0.001. Statistical analyses of Proteome Profiler and drug screening are described in the specific Supplementary Methods sections. Serial transplantation/limiting dilution assays were analyzed by extreme limiting dilution analysis (ELDA) software [30]. Principal Component Analysis was applied so to get a synthetic index of potency in the two tests (PC1) together with an independent index (PC2) of differential activity. The dose/effect and relative potency relationship of low-toxicity compounds was estimated in terms of LD50 by means of a logarithmic dose/effect relation model Vitality = a – b*log(dose).

## Results

### Workflow for CTCs-derived organoid generation from an orthotopic/metastatic mouse xenograft model

**Fig. 1:**
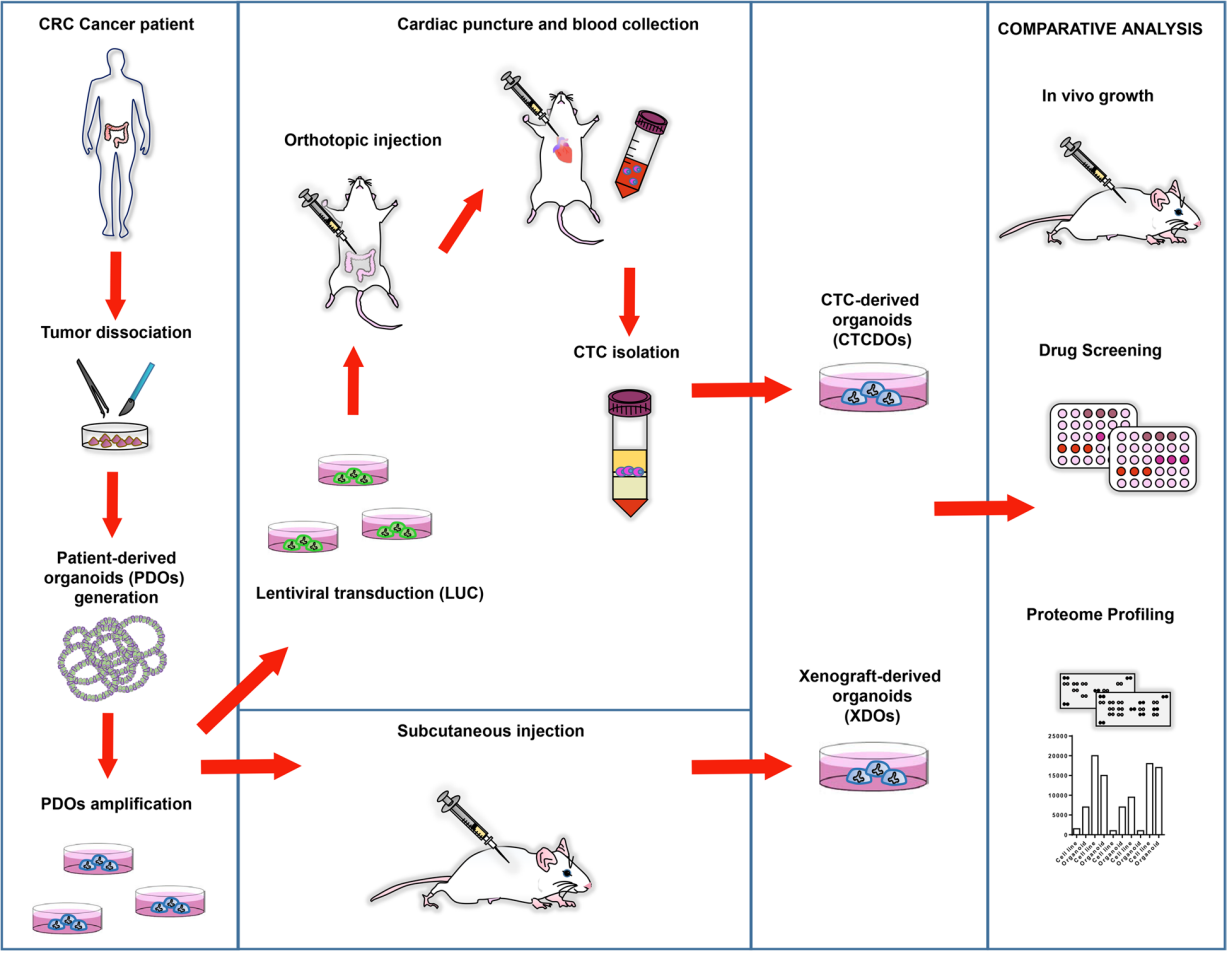
Establishment of organoid cultures generated from CRC patients (PDOs), subcutaneous xenografts (XDOs) or CTCs (CTCDOs). PDOs were directly generated from a colon adenocarcinoma surgically removed from a CRC patient. To obtain CTCDOs, PDOs were expanded, transduced with a luciferase-(LUC) encoding vector and orthotopically injected in the colon of immunocompromised mice. CTCs were then isolated from mouse peripheral blood collected by cardiac puncture and used to produce CTCDOs. XDOs were generated from subcutaneous xenografts and used a control for comparative in vitro and in vivo analyses

**Fig. 2:**
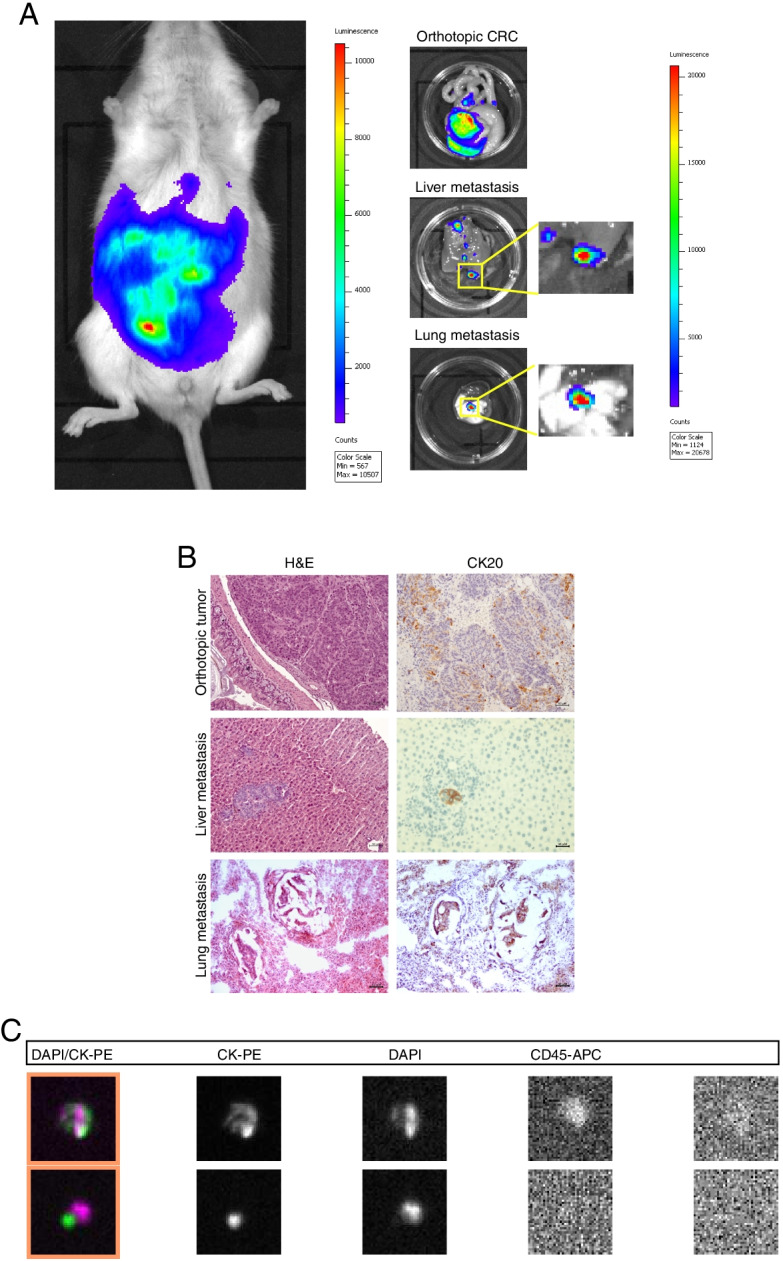
The orthotopic CRC xenograft model recapitulates the physiological metastatic process. **A** LUC-expressing PDOs were orthotopically injected into the colon of NSG mice, which were monitored for tumor growth by bioluminescent imaging (left panel). Upon sacrifice, orthotopic tumors and metastases were removed and quantified by bioimaging (central panels and right graph). **B** Paraffin-embedded section of PDOs-derived orthotopic tumor, liver and lung metastases were stained with Hematoxylin/Eosin (H&E, left panels) and cytokeratin-20 (CK20, right panels), 20x magnification. **C** Representative images of EpCAM-positive/cytokeratins (CK)-positive circulating tumor cells detected in mice whole blood. Abbreviations: CK-PE, cytokeratin (green); PE, phycoerythrin; DAPI; 4′,6-diamidino-2-phenylindole (violet); CD45-APC; APC, allophycocyanin

The workflow for CTCDOs generation is illustrated in Fig. 1. Tumor tissue derived from the surgical resection of a stage IV colonic adenocarcinoma was dissociated into single cells and used to generate patient-derived organoids (PDOs) according to the method originally created by Sato and coworkers [29]. MSI status and mutational profile of the tumor of origin are reported in Additional file 5, Fig. S1A and Fig. S1B. Short tandem repeat (STR) analysis of the primary tumor and PDOs confirmed their common cellular origin (data not shown). PDOs were also validated for their capacity to generate subcutaneous tumor xenografts that displayed the same histological structure of the patient’s tumor of origin (Additional file 5: Fig. S1C). The efficiency of PDOs generation from surgical specimens in our hands is around 90%, while subcutaneous PDOs engraftment has a 100% success rate. PDOs were then transduced with a LUC-encoding lentiviral vector to allow bioluminescence-based monitoring of tumor growth, and subsequently inoculated orthotopically into the colon of immunocompromised NSG mice. Orthotopic CRC xenografts spontaneously generate liver and lung metastases, thus recapitulating the metastatic process of human CRC (Fig. 2A and Additional file 6: Fig. S2) [31, 32]. The procedure of PDOs orthotopic xenografting in our model has an efficiency of 70-80%. Histological structure and Cytokeratin 20 (CK20) staining of orthotopic xenografts and of the deriving hepatic and pulmonary metastases confirmed their CRC origin (Fig. 2B). Analogously to human tumors, orthotopic CRC xenografts release CTCs, which were detected in the mouse peripheral blood with the CellSearch® platform as EpCAM^+^/CK (cytokeratins)^+^/mCD45^−^ cells (Fig. 2C). In order to collect CTCs, mouse blood was harvested from orthotopic/metastatic xenografts by cardiac puncture, and then CTCs were isolated by density centrifugation and negative selection with murine anti-CD45 antibody (mCD45) (see Methods and Additional file 1: Supplementary Methods sections). Of note, a minority of mice bearing orthotopic-metastatic xenografts had detectable CTCs (as described in the following section) and thus CTCs isolation from mouse peripheral blood represents the limiting step of the workflow illustrated in Fig. 1. CTCs were then cultivated to generate CTCDOs. Alternatively, PDOs were used to obtain subcutaneous xenografts and xenograft-derived organoids (XDOs) that were used as a control for subsequent experiments in comparison with CTCDOs, to rule out the possibility that the effects seen in CTCDOs may be due to cell passage in mouse.

### Generation and phenotypic characterization of CTC-derived organoid cultures

**Fig. 3:**
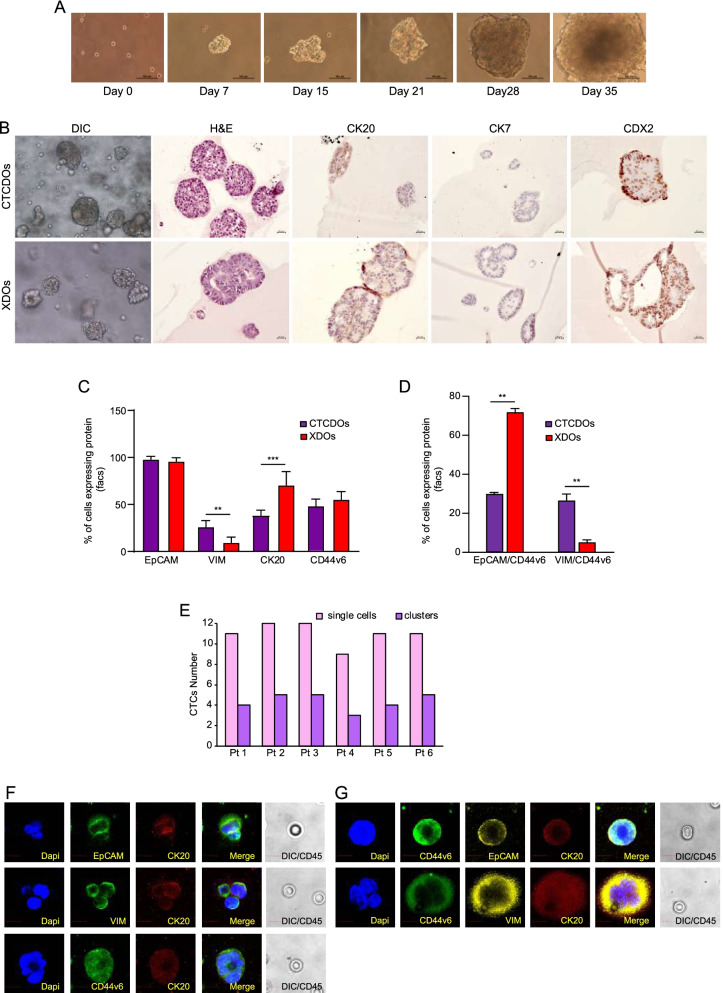
Generation and phenotypic characterization of CTCDOs cultures. **A** Time course images of CTCDOs culture. **B** Images of paraffin embedded sections obtained from CTCDOs (upper panels) and XDOs (lower panels). From left to right: Differential interference contrast (DIC), H&E staining, cytokeratin-20 (CK20), cytokeratin-7 (CK7), Caudal Type Homeobox 2 (CDX2). **C** Flow cytometry experiments showing the percentage of EpCAM, Vimentin (VIM), CK20 and CD44v6 positive cells in CTCDOs (purple bars) and XDOs (red bars) cultures. Values are obtained as the ratio between positive cells and total cells multiplied by 100, and represent the mean ± SD of three independent experiments performed with technical replicates. **D** Percentage of EpCAM^+^/CD44v6^+^, VIM^+^/CD44v6^+^ cells in CTCDOs (purple bars) and XDOs (red bars). Values are obtained as the ratio between positive cells and total cells multiplied by 100, and represent the mean ± SD of three independent experiments performed with technical replicates. **E** CTCs enumeration performed on the whole ScreenCell® filters obtained from each patient (Pt 1-6). Aggregates containing ≥3 CD45-negative cells were considered as CTCs clusters. Single cells: pink bar, clusters: violet bar. **F** CTCs isolated from the peripheral blood of CRC patients and stained with EpCAM (green)/CK20 (red); VIM (green)/CK20 (red); CD44v6 (green)/CK20 (red); (**G**) CTCs isolated from the peripheral blood of CRC patients and stained with CD44v6 (green)/EpCAM (yellow)/CK20 (red) or CD44v6 (green)/VIM (yellow)/CK20 (red). Nuclei were stained with DAPI. CD45 staining was performed with diaminobenzidine (DAB) and detected on differential interference contrast (DIC) images, appearing negative on CTCs and positive on blood cells (Additional file 6: Fig. S2D). Images are representative of at least three CTCs isolated from three different patients. Magnification 60x, 5x zoom. Bar 10 μM

In order to obtain CTCDOs, we generated orthotopic CRC xenografts in 8 mice. We destined the blood of 4 mice to the CellSearch® analysis shown in Fig. 2C, which detected CTCs in 1/4 mice (two CTCs in one mouse and none in the other three mice). The remaining four mice were used directly for CTCs isolation, without prior CTCs detection. Mouse blood was processed through density centrifugation and negative selection with mCD45, and the resulting pellets were placed in organoid culture. One sample out of four generated CTCDOs, which were used for further analyses (Fig. 3A). CTCDOs showed a CK20^+^/CDX2^+^/CK7^−^ phenotype typical of CRC (Fig. 3B). CTCDOs and XDOs were then analyzed by flow cytometry for the expression of markers associated to the epithelial/mesenchymal state (EpCAM, Vimentin), to intestinal differentiation (CK20) and to CRC stemness/metastatic ability (CD44v6) (Fig. 3C and Additional file 7: Fig. S3A-C). While EpCAM expression was comparable between CTCDOs and XDOs, Vimentin was more expressed in CTCDOs in line with a shift towards a mesenchymal state, while CK20 was decreased possibly reflecting a less differentiated state (Fig. 3C). The absolute levels of CD44v6 were comparable between CTCDOs and XDOs (Fig. 3C). To gain more insights into CD44v6 distribution on CTCDOs and XDOs, we analyzed CD44v6 expression in organoid subpopulations characterized by a prevalent epithelial (EpCAM^+^) or mesenchymal (Vimentin^+^) state. Interestingly, we observed that in CTCDOs the expression of CD44v6 was equally distributed among EpCAM^+^ cells and Vimentin^+^ cells, indicating their hybrid EMT state and high heterogeneity (Fig. 3D). By contrast, CD44v6 expression in XDOs was found prevalently on EpCAM^+^ cells (Fig. 3D). To investigate whether CTCDOs paralleled CRC CTCs in terms of phenotypic features, we isolated CTCs from the peripheral blood of six CRC patients (details in Additional file 2: Table S1) with the ScreenCell® technology in order to perform immunofluorescence analyses. The number of cells (both single CTCs and CTCs clusters) detected in the six patients used for this analysis is reported in Fig. 3E. Then, immunofluorescence analysis was performed on CTCs, confirming the expression of EpCAM, Vimentin and CD44v6 on CK20^+^/CD45^−^ CTCs (Fig. 3F). Blood cells stained positive for CD45 (Additional file 7: Fig. S3D) and were excluded from the analyses. Moreover, double staining for EpCAM/CD44v6 and Vimentin/CD44v6 confirmed the presence of putative CD44v6^+^ metastatic cells characterized by a combined epithelial (EpCAM^+^) and mesenchymal (Vimentin^+^) phenotype, providing further evidence to CTCs heterogeneity (Fig. 3G).

### CTC-derived organoids exhibit increased aggressiveness, stem cell content and migratory ability

**Fig. 4:**
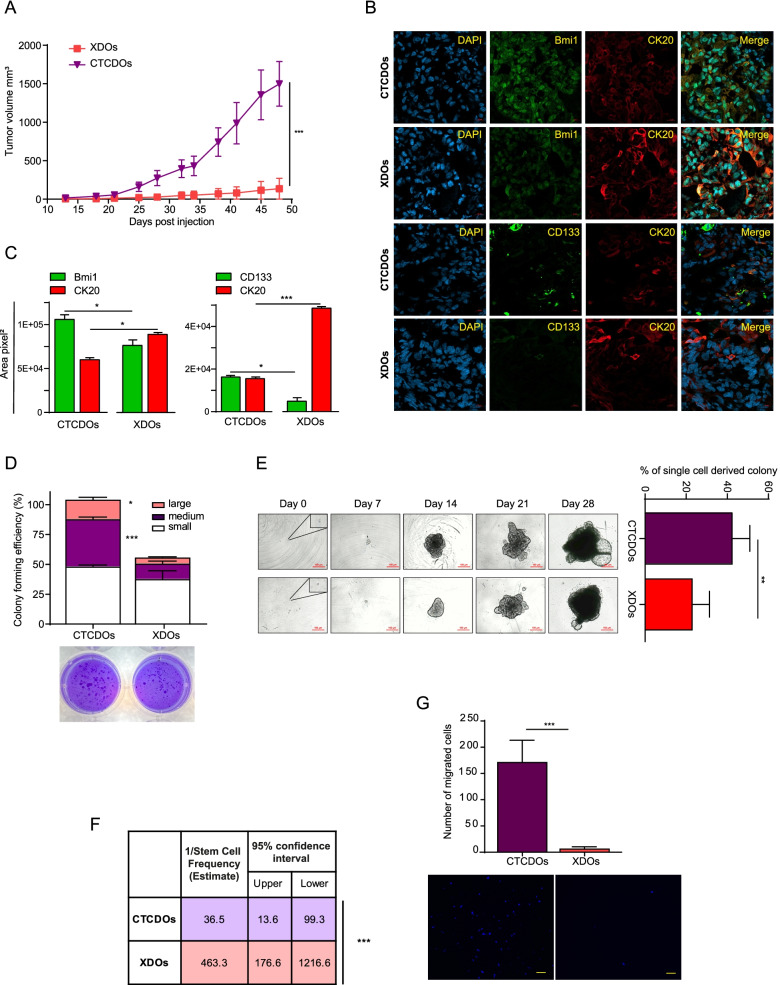
CTCDOs display increased aggressiveness, stem cell content and migratory ability. **A** Volume of subcutaneous tumor xenografts derived from XDOs (red line/squares) or CTCDOs (purple line/triangles). Mean ± SEM, 5 tumors per group. *P < 0.05 and **P < 0.01 by unpaired Student’s t test with Welch’s correction. **B** Representative images of immunofluorescence staining (40x magnification) of Bmi1, CK20 and CD133 in xenograft sections obtained from tumor xenografts of Fig. 4A; nuclei were stained with DAPI. **C** Quantification of Bmi1, CK20 and CD133 performed on xenograft sections obtained from tumor xenografts of Fig. 4A, 5 fields/section. **D** Self-renewal capacity of cells isolated from xenografts of Fig. 4A, evaluated as colony formation in semisolid culture and expressed as normalized colony size/percentage over plated cells. Values represent the mean ± SD of three technical replicates. *P* < 0.05; ***P < 0.001 by unpaired Student’s t test. **E** Single-cell assay performed with CTCDOs- and XDOs-derived cells (right) and time course images of CTCDOs and XDOs cultures (left). 10x magnification, bar 100 μm. Values represent mean ± SD of three independent experiments. **P < 0.01 by unpaired Student’s t test. **F** Tumor initiating cells content of CTCDOs and XDOs cultures was evaluated through serial transplantation/limiting dilution assays and quantified with the extreme limiting dilution analysis (ELDA) software. Five mice were used for each dilution point. ***P < 0.001. **G** Invasion/migration assay performed with CTCDOs and XDOs. The upper panel graph indicates the number of migrated CTCDOs and XDOs, while the lower panels show representative images of nuclei of CTCDOs- and XDOs migrated cells stained with DAPI (10x magnification). Values represent mean ± SD of three independent experiments. ***P < 0.001

Previous studies showed that CTCs are characterized by stem cell properties and tumor-forming capacity [33, 34]. Therefore, we performed functional analyses to compare stemness and tumorigenicity in CTCDOs as compared to XDOs. First, we inoculated subcutaneously the same number of cells derived from XDOs or CTCDOs and we monitored subsequent tumor growth. Cells derived from CTCDOs generated more aggressive tumors as compared to XDOs-derived cells (Fig. 4A). Accordingly, we found a higher expression of the stem cell marker CD133 [35, 36] and of the self-renewal marker Bmi1 [37] in tumor xenografts sections derived from CTCDOs (Fig. 4B-C), while the differentiation marker CK20 was expressed at lower levels as compared to XDOs-derived xenografts. Cells isolated from tumor xenografts were placed in semisolid culture in order to evaluate their colony-forming ability. In such conditions, cells isolated from CTCDOs-derived tumors gave rise to a significantly higher number of colonies (particularly medium and large colonies) indicating an increased clonogenic and replicative capacity (Fig. 4D). This observation was confirmed by a liquid culture single-cell assay comparing the clonogenic capacity of cells derived from dissociated CTCDOs or XDOs (Fig. 4E). Then, we aimed to assess the relative amount of tumor-initiating cells (TICs) in CTCDOs or XDOs through an in vivo limiting dilution/serial transplantation assay. The relative TICs content calculated by the extreme limiting dilution assay (ELDA) [30] was 1/36,5 in CTCDOs and 1/463,3 in XDOs (Fig. 4F), providing further support to the enhanced stem cell content of CTCDOs. Finally, we compared the migratory and invasive capacity of CTCDOs and XDOs through the transwell migration/invasion assay. While XDOs were virtually unable to invade Matrigel and migrate towards the chemoattractant medium, CTCDOs exhibited a marked invasive and migratory capacity, thus reproducing a functional feature of metastatic cancer cells (Fig. 4G).

### CTC-derived organoids have a distinctive therapy response profile and are more sensitive to drugs affecting the Survivin pathway

**Fig. 5:**
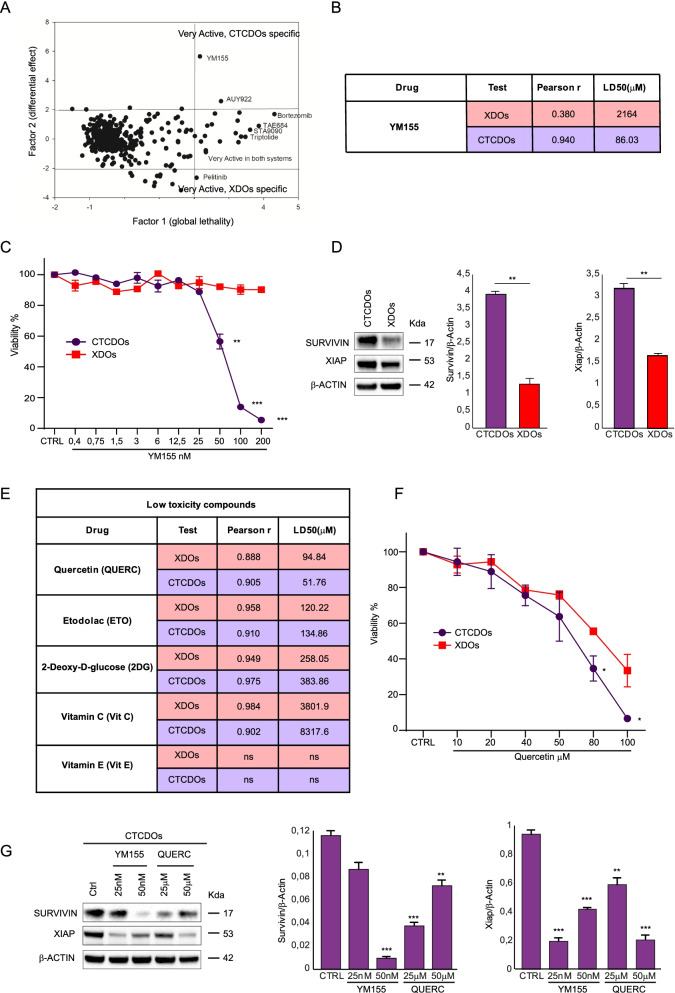
CTCDOs show a distinctive therapy response profile. **A** Principal component analysis (PCA) of cell viability assay performed with anti-cancer compounds (detailed in Additional file 3: Table S2) highlighting drugs very active on CTCDOs and XDOs, respectively. Drugs were used at a 200 nM concentration. **B** Schematic representation of YM155 potency (LD50, μM) and dose/effect relationship (Pearson r) in XDOs (red lines) and CTCDOs (purple lines). Doses of YM155 are reported in Fig. 5C. **C** Cell viability of CTCDOs (purple line/circles) and XDOs (red line/squares) treated with YM155 at the indicated concentrations. Values represent the mean ± SD of three independent experiments. **P < 0.01 and ***P < 0.001 by unpaired Student’s t test. **D** Left: immunoblot analysis of Survivin and XIAP on whole lysates of CTCDOs and XDOs. β-actin was used as a loading control. Right: quantification of immunoblot experiments (three replicates). **E** Schematic representation of low toxicity compounds potency (LD50, μM) and dose/effect relationship (Pearson r going from 0.88 to 0.98) in XDOs (red lines) and CTCDOs (purple lines). Doses of low toxicity compounds are reported in Additional file 3: Table S2. **F** Cell viability of CTCDOs- (purple line/circles) and XDOs (red line/squares) treated with quercetin (QUERC) at the indicated concentrations. Values represent mean ± SD of three independent experiments. *P < 0.05 and **P < 0.01 by unpaired Student’s t test. **G** Left: immunoblot analysis of Survivin and XIAP on whole lysates of CTCDOs treated for 6 days with the indicated concentrations of YM155 and quercetin. β-actin was used as a loading control. Right: quantification of immunoblot experiments (three replicates)

Few previous studies have investigated CTCs’ drug sensitivity profile [19, 38–40]. We performed a screening on CTCDOs and XDOs with a library of anti-cancer compounds and found that the two organoid cultures displayed a different drug sensitivity profile (Fig. 5A). In particular, XDOs were more sensitive to the EGFR inhibitor Pelitinib, whereas CTCDOs had an increased sensitivity to YM155, a drug targeting Survivin, and to the HSP90 inhibitor AUY922 (Luminespib). The LD50 of YM155 for XDOs and CTCDOs is shown in Fig. 5B. The differential response of CTCDOs and XDOs to YM155 was further confirmed by a dose-response cytotoxicity assay showing a highly significant difference starting from a dose of 50 nM (Fig. 5C). To gain more insights into the different effect of YM155 on CTCDOs and XDOs, we assessed the levels of Survivin and XIAP in the two cellular systems. As shown in Fig. 5D, CTCDOs expressed higher levels of both Survivin and XIAP, suggesting they may have an increased dependence on such pathway for their survival. The identification of a compound active on CTCs would be aimed at devising a therapeutic strategy for the prevention of metastatic disease. In this perspective, although YM155 (Sepantronium bromide) has successfully entered clinical trials, its pleiotropic mechanism of action may prevent its adoption for long-term therapeutic strategies involving non-metastatic patients. Therefore, in order to identify drugs potentially active on CTCs but with low toxicity on healthy tissues, we tested five additional compounds. Of these, three were chosen for their ability to inhibit EMT by interfering with metabolic pathways (Etodolac, 2-deoxy-D-glucose and vitamin C) [41] and two were chosen as multipurpose agents able to affect also the Survivin pathway (quercetin, vitamin E) [42]. The results of XDOs and CTCDOs challenging with different doses of the selected low toxicity compounds are shown in Fig. 5E, in Additional file 8 and in Fig. S4. While all the treatments with the exception of vitamin E were active (even if with a wide range of relative potency) with a statistically significant dose/effect, the only compound showing a significantly different effect (*P* < 0.05) between CTCDOs and XDOs was quercetin (Fig. 5E-F). Since both YM155 and quercetin have been shown to reduce cancer cell viability by decreasing Survivin protein levels [43, 44] we asked whether the same effect occurred in CTCDOs. An immunoblot analysis of Survivin and XIAP showed a decrease of both proteins upon treatment with either YM155 and quercetin, indicating this mechanism as a likely cause of CTCDOs death (Fig. 5G).

### Proteome profiling of CTC-derived organoids reveals expression of factors involved in stemness and stress response that are also expressed in CRC CTCs

**Fig. 6:**
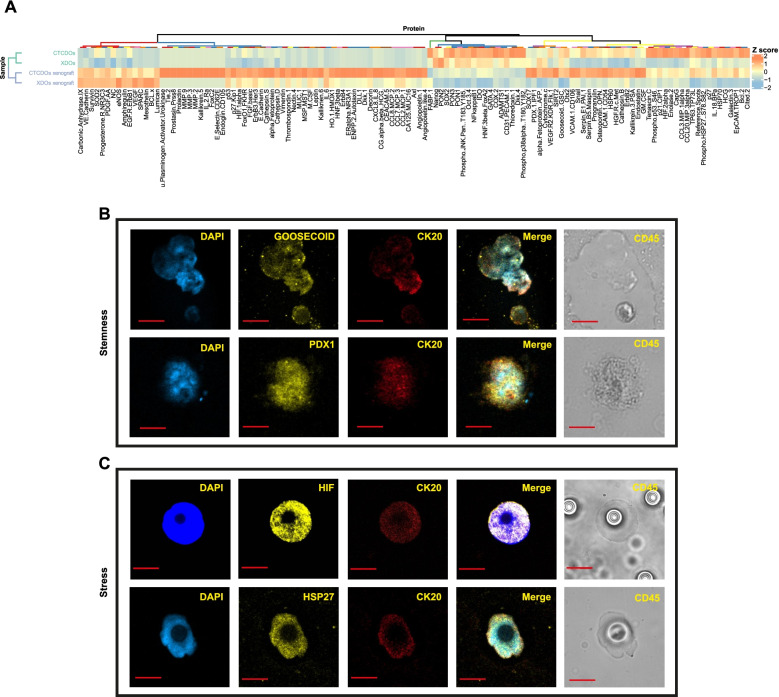
Proteome Profiler analysis of CTCDOs/CTCDOs-derived xenografts versus XDOs/XDOs-derived xenografts and selected protein validations on CRC CTCs. **A** Hierarchical clustering of Proteome Profiler results obtained on CTCDOs/XDOs and CTCDOs-derived tumor xenografts /XDOs-derived tumor xenografts *(n* = 2 pools of 3 tumors each). Clusters, identified for either antibodies or samples and based on optimal cut of dendrograms, are indicated by colored bars adjacent to dendrograms. The values represented by the heatmap correspond to normalized intensities of antibodies, standardized over the sample set analyzed (z score). A list of Proteome Profiler antibodies is reported in Additional file 4: Table S3. **B** Immunofluorescence analysis of stemness-associated factors Goosecoid and PDX1 on CTCs isolated from the peripheral blood of two CRC patients. **C** Immunofluorescence analysis of stress-associated factors HIF-1α and phospho-HSP27 on CTCs isolated from the peripheral blood of two CRC patients. Images are representative of at least three CTCs isolated from three different patients. Magnification 60x, 5x zoom, bar 10 μM

In order to generate a broader picture of proteins differentially expressed in CTCDOs and XDOs we analyzed the two systems with the Proteome Profiler Antibody Array®. In addition to CTCDOs and XDOs, we compared also subcutaneous xenografts derived from either CTCDOs or XDOs. Proteome analysis revealed an increased expression of factors involved in stemness (PDX1, Goosecoid, Oct3/4, SOX2, Delta-like protein/DLL1, Cited-2), stress response (Heat Shock Proteins HSP27, HSP60, HSP70, Hypoxia-Inducible Factors HIF-1α, HIF-2α), cell migration/invasion (Cathepsin D, Cathepsin S, Matrix Metalloproteinases MMP-2, MMP-3, MMP-9, c-Met) and EMT (Vimentin, Snail) in CTCDOs and/or in the deriving xenografts (Fig. 6A). Moreover, we observed an increased expression in CTCDOs of factors involved in antioxidant pathways (Manganese Superoxide Dismutase SOD2, Thioredoxin-1, Paraoxanase-1 and -2). The enhanced expression of HSPs, HIFs and antioxidant enzymes indicated that CTCDOs have an increased capacity to cope with oxidative stress, which has been recently recognized as a metastasis-promoting feature [45, 46] (Fig. 6A). A restricted number of endpoints was also validated by immunoblotting in CTCDOs, XDOs and in the respective xenografts (Additional file 9: Fig. S5). PDOs were also included in the analysis, providing a partial view of factor expression during the different stages of disease progression. Immunoblot analyses show that protein expression in PDOs was more similar to XDOs than to CTCDOs. Specifically, PDOs have lower levels of several factors (including B-cell lymphoma 2/Bcl-2, MMP2, Cathepsin D and Survivin) as compared to CTCDOs, suggesting that proteins related to tumor cell survival and aggressiveness increase during sequential stages of the disease. Notably, although xenografts tend to cluster together in the proteome profiler heatmap, CTCDOs-derived xenografts have a greater activation of signaling molecules involved in matrix remodeling, stress and EMT, even if compared to XDOs-derived xenografts. This observation substantiates the higher aggressiveness of CTCDOs-derived xenografts which cannot be attributed only to the in vivo passage but likely derives from the ability to recapitulates CTCs features. Finally, we ought to investigate whether selected factors that emerged from CTCDOs proteome profiling were also expressed in CTCs isolated from two CRC patients (Additional file 2: Table S1). Among stemness-related factors, we focused on the homeobox transcription factors Goosecoid and PDX1, as their expression is implicated in carcinogenesis and metastasis but was never detected in CTCs. In line with proteome profiling results, we found Goosecoid and PDX1 expression in CRC CTCs, possibly associated with a functional implication of these two factors in CRC metastasis (Fig. 6B). CTCs isolated from CRC patients also expressed high levels of stress-related proteins HIF-1α and phosphorylated HSP27 (Fig. 6C), suggesting a role in resistance to adverse microenvironmental conditions. CTCs observed positive for Goosecoid, PDX1, HIF1α and pHSP27 were respectively 3, 3, 12 and 3. Altogether, these observations indicate that CTCDOs recapitulate specific features of CRC CTCs such as the expression of stemness and stress response factors.

## Discussion

The analysis of CTCs isolated from the peripheral blood of cancer patients can provide important cues that may be useful to support clinical decision making. In particular, the possibility to perform a small-scale phenotypic drug screen would allow the identification of treatment strategies with increased tumor-killing ability. However, drug testing on CTCs requires the realization of ex vivo models based on CTCs expansion. CTCs-based cultures systems have been fulfilled in several tumors [6–20] and new methods to improve CTCs survival in vitro are currently being explored by many laboratories. Applying the organoid technology to CTCs is a promising strategy that combines the advantages of organoid cultures (ability to reproduce the architecture and function of original tissue, high predictive power in preclinical studies) with the enormous potential of CTCs for precision oncology. So far, the direct derivation of organoids from CTCs has been achieved in prostate cancer [39, 47], ovarian cancer [48, 49] and head and neck cancer [50]. However, organoid generation from CTCs remains a challenging task due to CTCs rareness. A possible solution has been implemented by Mout and coworkers by collecting high CTCs numbers for organoid culture from the diagnostic leukapheresis of prostate cancer patients [39]. In this work we describe the generation and characterization of organoid cultures derived from CTCs isolated from an orthotopic/metastatic mouse model of CRC. The CTCDOs experimental model presented in this study provides several advantages as compared to other CTCs culture systems. First, organoids allow tumor cells to adopt a more physiological architecture, behavior and differentiation hierarchy as compared with other experimental systems [51], thus resulting in a more faithful reproduction of CTCs features. In fact, CTCDOs showed several molecular and functional features of CTCs including increased aggressiveness, tumor-forming capacity, migratory/invasive ability, heterogeneity and expression of proteins involved in stemness, stress response and EMT [46]. Secondly, the CTCDOs model bypasses the need to collect sufficient numbers of CTCs from the peripheral blood of cancer patients. Third, CTCDOs can be established early after diagnosis starting from surgical tumor specimens, theoretically allowing the generation cells with metastatic features even before the onset of metastatic disease. Due to the technical challenges in CTCs isolation and expansion, few studies have previously investigated CTCs drug sensitivity profiles. Such studies were performed on CTCs-derived cultures of prostate cancer [39, 40], breast cancer [19] and small cell lung cancer [38]. To our knowledge, this is the first time that a library of anticancer compounds is tested on CTCs-derived organoid cultures, thus providing new insights on CTCs patterns of drug sensitivity. Specifically, we found an increased cytotoxic efficacy of Survivin and HSP90 inhibitors in CTCDOs as compared to XDOs. These results prompted us to further search for compounds with low toxicity that would preferentially target CTCDOs by decreasing Survivin levels [42]. Accordingly, we observed that CTCDOs were significantly more sensitive to quercetin which, similarly to YM155, was able to decrease Survivin and XIAP levels. Finally, we produced a comprehensive profile of proteins involved in stemness, stress response and tumorigenesis in CTCDOs and CTCDOs-derived xenografts. In line with CTCDOs enhanced aggressiveness and tumor-forming capacity, we found an increased expression of several factors implicated in stemness maintenance both in normal and malignant tissues such as PDX1, Goosecoid, Oct3/4, SOX2, DLL1, Cited-2. Among these, the homeobox transcription factors Goosecoid and PDX1 have been previously shown to be involved respectively in EMT/metastasis of breast and liver carcinomas [52, 53] and in pancreatic tumorigenesis and metastasis formation [54, 55]. Goosecoid and PDX1 expression was found in CTCs isolated from CRC patients, suggesting that these factor may play a role also in CRC metastatic process. Finally, the high expression of factors involved in migration/tissue invasion (Cathepsin D, Cathepsin S, MMP-2, MMP-3, MMP-9, c-Met), stress response (HSP27, HSP60, HSP70, HIF-1α, HIF-2α) and antioxidant activity (SOD2, Thioredoxin-1, Paraoxanases) further confirms the metastatic propensity of CTCDOs. In particular, the enhanced antioxidant capacity has been recently indicated as a key feature of metastatic dissemination [45, 46], further substantiating the similarity between CTCDOs and metastasis-competent CRC cells. These results, if confirmed by further studies on a larger number of patients, will be useful to characterize new druggable targets in CTCs. In summary, the results presented in this study contribute to depict a picture of CTCs as cells endowed with particular plasticity, stemness, adaptability and aggressiveness. This view is in line with the finding, emerged in the last years, that CTCs disseminate early during cancer progression and may reside in distant organs for many years as dormant cells that retain metastatic potential [56]. Unravelling CTCs survival mechanisms and drug vulnerabilities carries important clinical implications, possibly allowing the development of strategies for metastasis prevention.

## Conclusions

Our results provide preliminary evidence that CTCDOs may provide a suitable model to investigate CTCs molecular features, to identify timely strategies for metastasis prevention and to test personalized treatments. Future applications of the organoid technology to CTCs may open new perspectives by providing unprecedented insights onto the metastatic process, by allowing the detection of new CTCs biomarkers, therapeutic targets and chemoresistance mechanisms.

## Supplementary Information

**Additional file 1:.** Supplementary Methods.

**Additional file 2: Table S1.** Information of CRC patients used to isolate CTCs. NA: Not Available; CT: Chemotherapy.

**Additional file 3: Table S2.** List of compounds used in the screening: drug name, targets Selleckchem catalogue number and doses are reported. Anti-cancer compounds (sheet 1) have been used at a 200 nM concentration, whereas low toxicity compounds (sheet 2) have been used at the indicated doses in order to identify the LD50 value reported in Fig. 5E.

**Additional file 4: Table S3.** List of proteins and raw endpoint values relative to the Proteome Profiler Arrays shown in Fig. 6A.

**Additional file 5: Figure S1.** MSI status/mutational profile of patient’s CRC and xenograft validation. (A) WES analysis was performed on the surgical sample used for organoid generation, allowing the estimation of mutation rates (hypermutated: more than 10 mutations per Megabase). High-grade microsatellite instability (MSI) was also detected. Both Non-Silent (blue) and Silent (orange) somatic variants are reported. (B) OncoPrint showing functionally relevant intragenic lesions in recurrently mutated genes. Half boxes and full boxes represent heterozygous and homozygous variants, respectively; colors are used to specify the type of mutation. Stars indicate germline mutations; multiple hits affecting the same gene are indicated by numbers. (C) Hematoxylin/Eosin staining of the primary patient’s tumor and of PDOs-derived subcutaneous tumor xenograft showing comparable histological structure. Magnification 20x.

**Additional file 6: Figure S2.** Growth and metastatization of orthotopic tumor xenografts recorded by bioimaging. Representative images of LUC-expressing PDOs orthotopically injected into the colon wall of NSG mice and monitored by bioluminescent imaging (IVIS imaging system) at different time points.

**Additional file 7: Figure S3.** CTCDOs phenotypic characterization. (A) Flow cytometry of purified CTCDOs stained for human EpCAM and mouse CD45 expression. (B) Representative flow cytometry plots performed on XDOs and CTCDOs for the expression of: EpCAM, VIM, CK20, CD44v6 shown in Fig. 3C. (C) Representative flow cytometry plots performed on XDOs and CTCDOs for the expression of CD44v6/EpCAM and CD44v6/Vimentin Fig. 3D. (D) Representative confocal images of CD45-positive (hematopoietic) cells present on ScreenCell® filters. Cells were stained with CD45/DAB (appearing as the dark staining in the differential interference contrast/DIC brightfield), CD44v6 (green), EpCAM (yellow) and CK20 (red). Nuclei were counterstained with DAPI. Magnification 60x, 5x zoom, bar 10 μM.

**Additional file 8: Figure S4.** Linear regression of the effect of low toxicity compounds on CTCDOs and XDOs. Schematic representation trough linear regression of low toxicity compounds described in Fig. 5D and used at different indicated doses specified in Additional file 3: Table S2 (upper panel); detail of endpoints contained in the red square (lower panel).

**Additional file 9: Figure S5.** Selected protein validations on CTCDOs, XDOs, PDOs and CTCDOs/XDOs-derived xenografts. Left: immunoblot analysis of E-Cadherin, Vimentin, p21, P53, PON2, pHSP27 (Ser78), HIF-1α, Sirt2, Bcl-2, MMP2, Cathepsin D and Survivin on whole lysates of CTCDOs, XDOs, PDOs and CTCDOs/XDOs-derived xenografts (reported as CTCDOs xeno and XDOs xeno). β-actin and GAPDH were used as a loading control. Right: quantification of immunoblot experiments.

## Data Availability

All data generated or analysed during this study are included in this published article and its supplementary information files.

## Abbreviations

CTC: Circulating tumor cells
CRC: Colorectal cancer
CTCDOs: CTCs-derived organoids
EMT: Epithelial-mesenchymal transition
PDX1: Pancreatic Duodenal Homeobox 1
XDOs: Xenograft-derived organoids
PDOs: Patient-derived organoids
NSG: NOD.Cg-Prkdc^scid^ Il2rg^tm1Wjl^/SzJ
LUC: Luciferase
DAPI: 4′,6-diamidino-2-phenylindole
SD: Standard Deviation
SEM: Standard Error of Mean
ELDA: Extreme limiting dilution analysis
PCA: Principal Component Analysis
LD50: Lethal dose 50
CK: Cytokeratins
EpCAM: Epithelial cell adhesion molecule
CD45: Leucocyte common antigen
MSI: Microsatellite instability
STR: Short tandem repeat
CDX2: Caudal Type Homeobox 2
CD44v6: Variant 6 of the cell surface glycoprotein CD44
CD133: Prominin-1
Bmi1: Polycomb complex protein
TIC: Tumor-initiating cells
EGFR: Epidermal Growth Factor Receptor
YM155: Sepantronium bromide
HSP: Heat shock proteins
AUY922: Luminespib
XIAP: X-linked inhibitor of apoptosis protein
Oct3/4: Octamer binding transcription factor 3/4
SOX2: SRY-Box Transcription Factor 2
DLL1: Delta-like protein
Cited-2: Cbp/P300 Interacting Transactivator With Glu/Asp Rich Carboxy-Terminal Domain 2
HIF: Hypoxia-inducible factor
MMP: Matrix metalloproteinase
c-Met: Tyrosine-protein kinase Met
SOD2: Manganese superoxide dismutase
p21Cip1: Cyclin-dependent kinase inhibitor 1
p53: Tumor protein P53
PON2: Paraoxonase 2
SIRT2: Sirtuin 2
Bcl-2: B-cell lymphoma 2
GAPDH: Glyceraldehyde-3-Phosphate Dehydrogenase
