# Supplementary material for: An organoid model of colorectal circulating tumor cells with stem cell features, hybrid EMT state and distinctive therapy response profile
Source: J Exp Clin Cancer Res. 2022 Mar 8;41:86. doi: 10.1186/s13046-022-02263-y (PMC8903172; doi:10.1186/s13046-022-02263-y)
Supplement: Supplementary file 1 — Additional file 1:. Supplementary Methods. [file 13046_2022_2263_MOESM1_ESM.docx]

**Supplementary Methods**

**Generation and validation of patient-derived organoids (PDOs), xenograft-derived organoids (XDOs) and circulating tumor cell-derived organoids (CTCDOs)**

Colorectal cancer (CRC) specimens were obtained from patients undergoing surgical resection upon informed consent and approval by the Sapienza-Policlinico Umberto I Ethical Committee (RIF.CE: 4107 17/10/2016). PDOs used in this study were obtained from a 69 years old male CRC patient undergoing surgery for a G2 stage IVA left colon tumor. Tissue samples were collected by a pathologist immediately after surgery, quickly washed 2–3 times in cold phosphate buffered saline (PBS) and then transferred in Dulbecco’s modified Eagle’s medium (DMEM; Thermo Fisher, Foster City, CA, USA) containing 3% penicillin-streptomycin-amphotericin B solution (Thermo Fisher) until processing. For tissue dissociation, CRC samples were first washed 3–4 times in PBS, then cut by forceps and/or scalpel in pieces of approximately 0.5 mm or smaller. Fragments were further washed twice by centrifugation at 150 g for 3 min, then incubated in Tryple Express 1X (Thermo Fisher) for 30 min at 37 °C under shaking. The cell suspension was then filtered through a 100 μm nylon mesh and washed by 2 further centrifugation steps in DMEM and resuspended in Matrigel^®^ (Growth Factor Reduced (GFR) Basement Membrane Matrix Corning, New York, USA). Matrigel^®^ containing cancer cell clusters were seeded into 24-well tissue culture plates (Thermo Fisher) and allowed to polymerize for 10 min at 37°C. The cancer cells were overlaid with 500 µL of colon cancer organoids culture medium as described by Sato et al. [1] supplemented with 20 ng/mL recombinant human EGF, 10 ng/ml human basic fibroblast growth factor (both from Peprotech, Rochy Hill, NJ, USA),10 nM Gastrin, 10 µM Y-27632, 10 µM SB202190 (Sigma-Aldrich, St. Louis, MO) and 500 nM A83-01 (Tocris Bioscience, Bristol, UK). Every 2 to 3 days, half of the culture medium was refreshed. The resulting PDOs cultures were then passaged weekly and used either to generate subcutaneous xenografts (that were processed as described above to obtain XDOs) or orthotopic xenografts. For CTCs isolation and CTCDOs generation, mice whole blood was centrifuged using a Lympholyte Cell separation media (#CL5020, Cedarlane Laboratories, Burlington, Canada) and CTCs isolated from the mononuclear cell layer were negatively selected by depleting mouse CD45-positive cells from a blood sample using ferromagnetic anti-CD45 coated beads following the manufacturer’s instructions (CD45 Microbeads, mouse #130-052-301, Miltenyi Biotec, Germany). The cell suspension was then washed by centrifugation step in DMEM, resuspended in Matrigel^®^ and cultured as described above to generate CTCDOs cultures. Genomic DNA was extracted from tumor tissue, organoids and patient-matched nontumor tissues with the DNeasy Mini Kit (Qiagen, Limburg, The Netherlands) and used for mutation analysis (tumoral and matched non tumoral tissue) and for Short Tandem Repeats (STR) analysis. The latter was performed with the AmpFlSTR Identifiler Plus Kit (Applied Biosystems) and used to generate a unique STR profile for each organoids cell line, which was used to monitor purity of the line over time and to confirm its matching with the original patient material. PDOs were also tested for their ability to generate xenografts reproducing the histology of the human tumor of origin. All organoid cultures were routinely tested for mycoplasma contamination with the PCR Mycoplasma Test Kit (Venor^®^GeM OneStep, Minerva Biolabs, Germany).

**Antibodies and Reagents**

Mouse monoclonal antibodies against human Prominin-1 (#130–090-423, Miltenyi Biotec, Germany), rabbit monoclonal anti-phospho-HSP27 (S78/S82) (MAB23141, R&D Systems), mouse monoclonal anti-human CK20 (#M7019, Dako, Agilent Technologies), goat polyclonal anti-human CK20 (#SC-17113, Santa Cruz Biotechnology), goat polyclonal anti-human Goosecoid (#AF4086, R&D Systems), mouse monoclonal anti-human CD44v6 (#BBA13, R&D Systems), polyclonal goat anti-human EpCAM (#AF960, R&D Systems), mouse monoclonal anti-human CD45 biotin (#130–098-551, Miltenyi Biotec), rabbit monoclonal anti-human BMI-1 (#6964S, Cell Signaling Technology), rabbit monoclonal anti-human PDX1 (#5679T, Cell Signaling Technology), rabbit monoclonal anti-human Vimentin (#5741S, Cell Signaling Technology), rabbit monoclonal anti-human CK20 (#13063S, Cell Signaling Technology), rabbit monoclonal anti-human HIF-1α (#36169, Cell Signaling Technology) were used for immunofluorescence. PE-conjugated anti-mouse CD45 (#561087, BD Biosciences), FITC-conjugated anti-human EpCAM (#F0860, Agilent Dako), APC-conjugated anti-human CD44v6 (#FAB36604, R&D Systems), FITC-coniugated anti-human Vimentin (#ab128507, Abcam) and rabbit monoclonal anti-human CK20 (#13063S, Cell Signaling Technology) were used for flow cytometry. The following secondary antibodies obtained from Thermo Fischer were used in the study: donkey anti-goat IgG Alexa Fluor®488-conjugated (#A11055), donkey anti-rabbit IgG Alexa Fluor®488-conjugated (#A21206), donkey anti-mouse IgG Alexa Fluor®488-conjugated (#A21202), donkey anti-goat IgG Alexa Fluor®647-conjugated (#A21447), donkey anti-mouse IgG Alexa Fluor®647-conjugated (#A31571), donkey anti-rabbit IgG Alexa Fluor®647-conjugated (#A31573), donkey anti-goat IgG Alexa Fluor®555-conjugated (#A21432), donkey anti-rabbit IgG Alexa Fluor®555-conjugated (#A31572). 4′,6-diamidino-2-fenilindole (DAPI, #D1306, 100 nM) and ProLong Gold Antifade (#P7481) was obtained from Thermo Fisher. Mouse monoclonal anti-human CK20 (#M7019, Dako, Agilent Technologies), mouse monoclonal anti-human CK7 (#M7018, Dako, Agilent Technologies) and and rabbit monoclonal anti-human CDX2 (#12306S, Cell Signaling Technology), were used for immunohistochemistry. Mayer’s haematoxylin (#MHS32) and Eosin (#HT110232) were from Sigma-Aldrich and used for H&E staining according to the manufacturer’s protocol. Rabbit monoclonal anti-human Survivin (#2808S), rabbit monoclonal anti-human XIAP (#14334S), rabbit monoclonal anti-human Vimentin (#5741), rabbit polyclonal anti-human Phospho-HSP27 (Ser78) (#2405), rabbit monoclonal anti-human HIF-1α (#36169), rabbit monoclonal anti-human Bcl-2 (#4223), rabbit polyclonal anti-human Cathepsin D (#2284) used for western blot were from Cell Signaling Technology while E-Cadherin (#610181) was from Becton Dickinson. Rabbit polyclonal anti-human MMP-2 (ab92536) was from Abcam (Cambridge, United Kingdom). Mouse monoclonal anti-human p21 (#sc-6246) and mouse monoclonal anti-human PON2 (#sc-373981) were from Santa Cruz Biotechnology (Dallas, Texas, USA). Rabbit polyclonal anti-human SIRT2 (#PA1-25472) and mouse monoclonal anti-human p53 (#MA5-12557) were from Thermo Fisher. Mouse monoclonal anti-β-Actin (#A5316) and rabbit polyclonal anti-GAPDH were from Sigma-Aldrich. Secondary mouse IgG, HRP-linked (#NA931, 1:4000) and rabbit IgG, HRP-linked (#NA934V, 1:4000) antibodies were from GE Healthcare Life Sciences.

**Animal procedures**

All animal procedures were performed according to the Italian National animal experimentation guidelines (D.L.116/92) upon approval of the experimental protocol by the Italian Ministry of Health’s Animal Experimentation Committee (DM n. 292/2015 PR 23/4/2015). 6-week-old female NOD.Cg-Prkdc^scid^ Il2rg^tm1Wjl^/SzJ (NSG) mice (The Jackson Laboratory) were used for experiments. For orthotopic colon wall injection, NSG mice were anesthetized with a mixture of ketamine (100 mg/kg) and xylazine (10 mg/kg) injected into the peritoneal cavity. 10^5^ cells obtained from dissociated PDOs and transduced with a luciferase (LUC)-expressing lentiviral vector were injected in the colon wall during open laparotomy and tumor formation was monitored with an IVIS imaging system (Perkin Elmer) once a week. For the isolation of CTCs, about 1000 µL of whole blood were drawn from anesthetized mice, by transthoracic cardiac puncture with a 1 mL syringe and collected into a K_2_EDTA BD microtainer blood collection tube. Mice were euthanized immediately after blood collection by cervical dislocation. For subcutaneous xenografts generation, 5 × 10^4^ dissociated cells obtained from PDOs or CTCDOs resuspended in 100 μl 1:1 PBS/Matrigel were injected in the flank of NSG mice. Tumors were measured twice weekly by an external digital caliper, and volumes were calculated using the following formula: π/6 x d2 x D, where d and D represent shorter and longer tumor measurements, respectively. Tumor growth was measured at the indicated time points. For the evaluation of stem cell content, secondary transplantations with serial cell dose dilution were performed. Primary xenografts were harvested, pooled and dissociated into single cells. For each pool, cells were injected into secondary mice at serial doses ranging from 10 to 10^3^. Five animals were used for each dilution point. Mice were recorded as negative when no graft was observed after 24 weeks from inoculation. Quantification of stem cell frequency was calculated by the ELDA software [2]. Animals were euthanized according to the national Animal Welfare Guidelines.

**CTCs detection in mouse peripheral blood**

Cardiac blood (about 1 mL), collected as described above, was immediately processed. CTC were identified by using reagents of CellSearch^®^ CTC kit, according to the protocol adapted from Veridex mouse/rat CellCapture Kit, as previously described [3, 4]. Briefly, CTC were first isolated using anti-EpCAM ferrofluid and Capture Enhancement Reagent, then incubated with Permeabilization Reagent, Nucleic Acid Dye, Staining Reagent, as well as rat monoclonal anti-mouse CD45-APC (#17-0451-82, Thermo Fischer). Samples were immune-magnetically separated, resuspended in 350 μl of dilution buffer and transferred to a CellSearch^®^ MagNest™ cartridge to be analyzed through CellSearch^®^ Analyzer II. An event was considered as a CTC when having round to oval morphology, a visible nucleus, positive staining for cytokeratins (CK) and negative staining for mCD45.

**CTCs isolation from CRC patients**

Peripheral blood samples were obtained from 6 patients with metastatic CRC at baseline before initiation of treatment at Policlinico Umberto I of Rome according to the protocol approved by Ethical Committee of Policlinico Umberto I of Rome (protocol n. 668/09, July 09, 2009; amended protocol 179/16, March 01, 2016) (Additional File **2**: Table S1). Each sample was collected into K_2_EDTA tube, stored at +4°C and processed within 3hrs from drawing. In order to isolate fixed CTC for cytological studies, Screencell^®^ Cyto kit (ScreenCell, Sarcelles, France) was employed, following the manufacturer’s instructions. Briefly, 3 mL of blood was diluted with 4 mL of ScreenCell Fixed Cells (FC2) dilution buffer containing red blood cell lysis and fixation buffer. After 8 min of incubation at room temperature, 7 mL of diluted blood was transferred into device tank and filtered under a pressure gradient using a vacutainer tube. After washing with 1.6 mL of PBS to remove red blood cells debris, the filter was left on absorbing paper to dry at room temperature and then mounted on a glass slide using Faramount Aqueous mounting medium (Dako, Agilent Technologies) and stored at -20°C or immediately analyzed. For each patient the blood filtration was carried out in duplicate. All participants signed an informed consent form before entering the study.

**Immunofluorescence staining of CTCs from CRC patients.**

For immunofluorescence, filters were hydrated with Tris-Buffered Saline (TBS) for 10 minutes and directly stained with anti-human biotinylated CD45 in order to eliminate hematopoietic cells as follow: filters were washed twice in TBS 0.002% Tween20, endogenous peroxidase activities were blocked using 0.03% hydrogen peroxide for 15 minutes in the dark, then the sections were incubated at room temperature for 1 hour 30 minutes with CD45 biotinylated antibody. Sections were then processed using streptavidin conjugated to horseradish peroxidase and substrate-chromogen solution both contained in UltraTek HRP Anti-Polyvalent DAB kit (#AMF080, Scytek), following manufacturer’s instructions. Samples were then incubated in a humid chamber overnight at 4°C with the following primary antibodies: anti-EpCAM, anti-Vimentin, anti-CD44v6, anti-phospho-HSP27, anti-CK20, anti-HIF1α, anti-PDX1, anti-Goosecoid. The filters were then washed twice in PBS and incubated with a mixture of appropriate secondary antibodies (Antibodies and Reagents section) for 45 minutes at room temperature in the dark. Nuclei were stained with DAPI for 15 minutes at room temperature. All antibodies were dissolved in PBS containing 3% bovine serum albumin (BSA), 3% fetal bovine serum (FBS), 0.001% NaN3 and 0.1% Triton X-100. Finally, the filters were mounted with Prolong-Gold Antifade (Thermo Fisher) on slides and analyzed using a Zeiss LSM900 confocal microscope or an Olympus FV1000 confocal microscope equipped with 60× oil immersion objectives.

**Immunofluorescence staining of xenograft-derived sections**

Tumour tissue samples were collected in Optimal Cutting Temperature (OCT compound), frozen on dry ice and stored at − 80 °C until further use. Five micrometers tissue sections were cut with a cryostat and mounted on coverslips. Tissue sections were fixed with 2% paraformaldehyde, permeabilized with 0.1% Triton X-100/PBS, quenched with 1 M glycine in PBS and incubated overnight at 4 °C with primary antibodies anti-CD133, anti-Bmi1 and anti-CK20. After washing in PBS, sections were incubated with a mixture of appropriate secondary antibodies (Antibodies and Reagents section) for 45 minutes at room temperature in the dark. Nuclei were counterstained with DAPI for 15 minutes at room temperature. All antibodies were dissolved in PBS containing 3% bovine serum albumin (BSA), 3% fetal bovine serum (FBS), 0.001% NaN3 and 0.1% Triton X-100. Slides were mounted with Prolong-Gold Antifade (Thermo Fisher) and analyzed using a Zeiss LSM900 Confocal microscope equipped with a 40× oil immersion objective.

**Western blotting**

Matrigel/organoid suspension was dissociated with TrypLE Express 1X (Thermo Fischer) and organoid pellets were lysed in the appropriate volume of lysis buffer: 1% NP40 lysis buffer (20 mM Tris HCl pH 7.2, 200 mM NaCl, 1% NP40), supplemented with protease inhibitor cocktail and phosphatase inhibitor cocktails I and II (all from Sigma-Aldrich). Lysate concentration was determined with the Bradford assay (Bio-Rad Laboratories, Hercules) and equal amounts of proteins were loaded on a 4–12% precast gel (Thermo Fisher) and transferred to nitrocellulose membranes (GE Healthcare Life sciences). Blots were blocked with TBST 5% nonfat dry milk (Bio-Rad Laboratories) and incubated overnight at 4 °C with primary antibodies (described in the Antibodies and Reagents section) diluted in TBST/BSA 5%, after 3 washes in TBST then incubated for 45 min with specific secondary HRP-conjugated antibodies diluted in TBST 5% nonfat dry milk. Images were taken and analyzed with Bio-Rad ChemiDoc Imagers (Bio-Rad Laboratories). For densitometry quantification immunoblot signals were acquired with ChemiDocMP (BioRad Laboratories) and the relative intensity was quantified with Image Lab software. Normalization was performed using β-Actin or GAPDH as reference.

**Drug screening and statistical analysis**

Anti-cancer compounds and low toxicity compounds were purchased from Selleck Chemicals, and listed in Additional File **3**: Table S2. Organoids were dissociated into single cells and plated in 30ul 1:1 Medium/Matrigel in 96 well plates (3,500 cells per well) in triplicate for 72 hours prior to drug treatment. Organoids were treated for 6 days in a humidified atmosphere at 37 °C, 5% CO_2_ and drug-containing medium was replaced every 72 hours. Cell viability was determined by CellTiter Glo 3D viability assay (Promega) with a DTX880 multimode microplate reader (Beckman Coulter). The correlation between the activities on the two test across all the library compounds was estimated by Pearson r correlation coefficient on the Lethality (100-Vitality) percent values. The statistical significance of the difference between the effects on the two tests on the entire data set was estimated by means of both non-parametric (Sign and Signed Rank tests) and parametric (Paired t-test) approaches. The bivariate space spanned by XDOs and CTCDOs lethality values having as statistical units the different drugs was analysed by a principal component analysis (PCA) approach. The bivariate character of the original space, together with the strong correlation between the two tests, force the PCA to give rise to a two component solution explaining the 100% of initial information. The first component (PC1) represents the by far most important (in terms of explained variance) component correspondent to a ‘size’ [5] component summarizing the average potency on the two tests and allowing for a straightforward activity ranking of the drugs. Being the principal components each other orthogonal by construction, the second component (PC2) quantifies the drug specificity (independent of the global potency) of action as for the two tests. [5]. The character of z-scores of the principal component scores allowed us to get a straightforward classification into active/inactive compounds on PC1 based on the PC1 > 2 threshold (correspondent to an effect greater than 2 SD from the entire data set mean). The above classification was contrasted with the presumptive mechanisms of action of the drugs by a chi-square test so obtaining an indication of the most promising target pathways. In order to generate a sensible ranking of Low Toxicity drug potencies, the LD50 of each drug was computed according to a general exponential model of Vitality decrease at increasing dose Vitality = k – a(log(dose). All the drugs with the only exception of Vitamin E that was inactive, showed a remarkable dose/effect relationship (Pearson r going from 0.88 to 0.98) so allowing to compute LD50 according to LD50 =10 ((k – 50)/a). In order to check for the presence of a statistical significant differential effect of the drugs in the two tests, giving the strong correlation between the two tests results on the entire data set we adopted a paired t-test strategy comparing the estimated results according to the linear model linking the two tests and the actual observed results.

**Immunohistochemistry**

Tissues and organoids were fixed in 4% paraformaldehyde (PFA) followed by dehydration, paraffin embedding, sectioning, and standard H&E staining. For IHC staining, the samples were incubated with primary antibodies anti-CK20, anti-CDX2 and anti-CK7 described in the Antibodies and Reagents section. The sections were subsequently incubated with secondary antibodies and visualised using the UltraTek HRP Anti-Polyvalent DAB (Scytek). Nuclei were counterstained with Mayer’s haematoxylin. Images were acquired on a Zeiss Axio Scope.A1 Microscope equipped with 20× and 40× objectives.

**Proteome Profiler Arrays**

Stem cell-related markers, cancer and stress-related proteins expression in organoids and xenograft-derived organoids were measured with Proteome Profiler Human Pluripotent Stem Cell Array Kit (#ARY010), Proteome Profiler Human XL Oncology Array (#ARY026) and Proteome Profiler Human Cell Stress Array Kit (#ARY018) all from R&D Systems according to the manufacturer’s instructions. Membranes were developed with ECL™ Prime Western Blotting Detection Reagent (#RPN2236, GE Healthcare Life Sciences) and images were acquired with ChemiDocMP (BioRad). Dot intensity was quantified with Western Vision Software`s HLImage++. Values were normalized to internal reference control. Analysis of Proteome Profiler data was performed by means of ‘R’ v4.03 [6] and RStudio v1.4 [7] using the following packages: base, openxlsx, tidyverse [8], RColorBrewer, circlize, dendextend and ComplexHeatmap [9]. Hierarchical clustering was performed using the complete method and the correlation distance matrix obtained from centered and scaled data. A detailed list of protein tested is available in Additional File **4**: Table S3.

**Flow cytometry**

Organoid-dissociated cells were labelled with primary antibodies: anti-mouse CD45, anti-human EpCAM, CD44v6, vimentin and CK20 for 1 hr on ice and, where necessary, with secondary antibodies for 30 min on ice (described in the Antibodies and Reagents section). Fluorescence intensity of labeled cells was evaluated with a FACSCanto flow cytometer. 10 μg/ml 7-aminoactinomycin D (Sigma-Aldrich) was always added for dead cell exclusion.

### Invasion/Migration assay

4 x 10^3^ cells obtained from dissociated XDOs or CTDOs were allowed to re-aggregate into organoids for 4 days in Matrigel® and then plated in Matrigel® into the upper wells of Boyden Chambers containing porous 8 μm diameter polycarbonate membranes (Costar Scientific Corporation) and suspended in 200 μl of non-supplemented organoids medium. Lower wells contained 500 μl of organoids medium supplemented with 20 ng/ml EGF and 10 ng/ml basic FGF. After 72 hours, the cells in the upper wells were removed, whereas the cells that migrated to the lower wells were fixed in 4% PFA, stained with DAPI in PBS/BSA 1% for 5 min and counted under a fluorescence Zeiss Axio Scope.A1 Microscope equipped with a 10× objective. The number of migrated cells was quantified with the software ZEN 2.6 (blue edition).

### Clonogenicity assay and single cell cloning

The clonogenic units present in xenograft-derived cells were assessed by plating 500 cells/ml per well in triplicate in 24-well plates containing a soft agar bilayer (0.3% top and 0.4% bottom layer; SeaPlaque Agarose; Cambrex). Cultures were incubated in humidified atmosphere at 37°C and 5% CO_2_ for 21 days. Colonies were stained with crystal violet (0.01% in 10:1 methanol to water), and counted under a light microscope. Data represent the percentage of colonies normalized to the number of cells plated. For single cell cloning, cells derived from either dissociated XDOs or CTCDOs were plated at single cells density by single-cell automated sorting on nine 96 well plates. After overnight culture wells were observed under a light microscope and wells with a single cell were recorded. After 30 days, clones generated from single cells were counted to calculate the percentage of colony forming cells.

**References**

[1] T. Sato, D.E. Stange, M. Ferrante, R.G. Vries, J.H. Van Es, S. Van den Brink, W.J. Van Houdt, A. Pronk, J. Van Gorp, P.D. Siersema, H. Clevers, Long-term expansion of epithelial organoids from human colon, adenoma, adenocarcinoma, and Barrett's epithelium, Gastroenterology, 141 (2011) 1762-1772.

[2] Y. Hu, G.K. Smyth, ELDA: extreme limiting dilution analysis for comparing depleted and enriched populations in stem cell and other assays, J Immunol Methods, 347 (2009) 70-78.

[3] J. Kitz, D. Goodale, C. Postenka, L.E. Lowes, A.L. Allan, EMT-independent detection of circulating tumor cells in human blood samples and pre-clinical mouse models of metastasis, Clin Exp Metastasis, 38 (2021) 97-108.

[4] L.E. Lowes, B.D. Hedley, M. Keeney, A.L.J.J.o.v.e.J. Allan, Adaptation of semiautomated circulating tumor cell (CTC) assays for clinical and preclinical research applications, J Vis Exp, (2014).

[5] P. Jolicoeur, J.E. Mosimann, Size and shape variation in the painted turtle. A principal component analysis, Growth, 24 (1960) 339-354.

[6] R.C. Team, A Language and Environment for Statistical Computing, R Foundation for Statistical Computing, Vienna, (2018).

[7] R. Team, RStudio: Integrated Development for R. RStudio, PBC, Boston, MA, (2020).

[8] H. Wickham, M. Averick, J. Bryan, W. Chang, L. D'Agostino McGowan, R. Francois, G. Grolemund, A. Hayes, L. Henry, J. Hester, M. Kuhn, T. Pedersen, E. Miller, S. Bache, K. Muller, J. Ooms, D. Robinson, D. Seidel, V. Spinu, K. Takahashi, D. Vaughan, C. Wilke, K. Woo, H. Yutani, Welcome to the Tidyverse, Journal of the Open Source Software, 4 (2019).

[9] Z. Gu, R. Eils, M. Schlesner, Complex heatmaps reveal patterns and correlations in multidimensional genomic data, Bioinformatics, 32 (2016) 2847-2849.
