# Supplementary figures and images for: An organoid model of colorectal circulating tumor cells with stem cell features, hybrid EMT state and distinctive therapy response profile
Source: J Exp Clin Cancer Res. 2022 Mar 8;41:86. doi: 10.1186/s13046-022-02263-y (PMC8903172; doi:10.1186/s13046-022-02263-y)

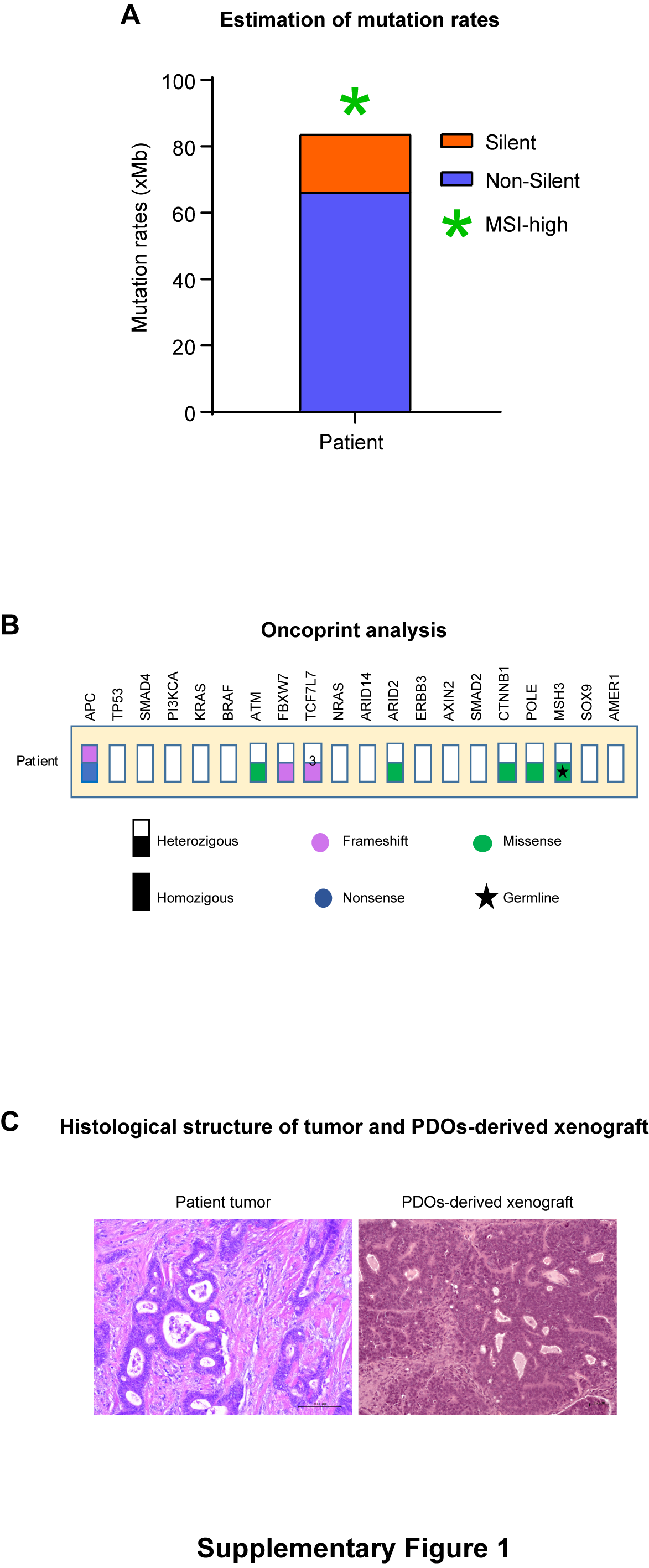

Supplement: Supplementary file 5 — Additional file 5: Figure S1. MSI status/mutational profile of patient’s CRC and xenograft validation. (A) WES analysis was performed on the surgical sample used for organoid generation, allowing the estimation of mutation rates (hypermutated: more than 10 mutations per Megabase). High-grade microsatellite instability (MSI) was also detected. Both Non-Silent (blue) and Silent (orange) somatic variants are reported. (B) OncoPrint showing functionally relevant intragenic lesions in recurrently mutated genes. Half boxes and full boxes represent heterozygous and homozygous variants, respectively; colors are used to specify the type of mutation. Stars indicate germline mutations; multiple hits affecting the same gene are indicated by numbers. (C) Hematoxylin/Eosin staining of the primary patient’s tumor and of PDOs-derived subcutaneous tumor xenograft showing comparable histological structure. Magnification 20x. [file 13046_2022_2263_MOESM5_ESM.tif]

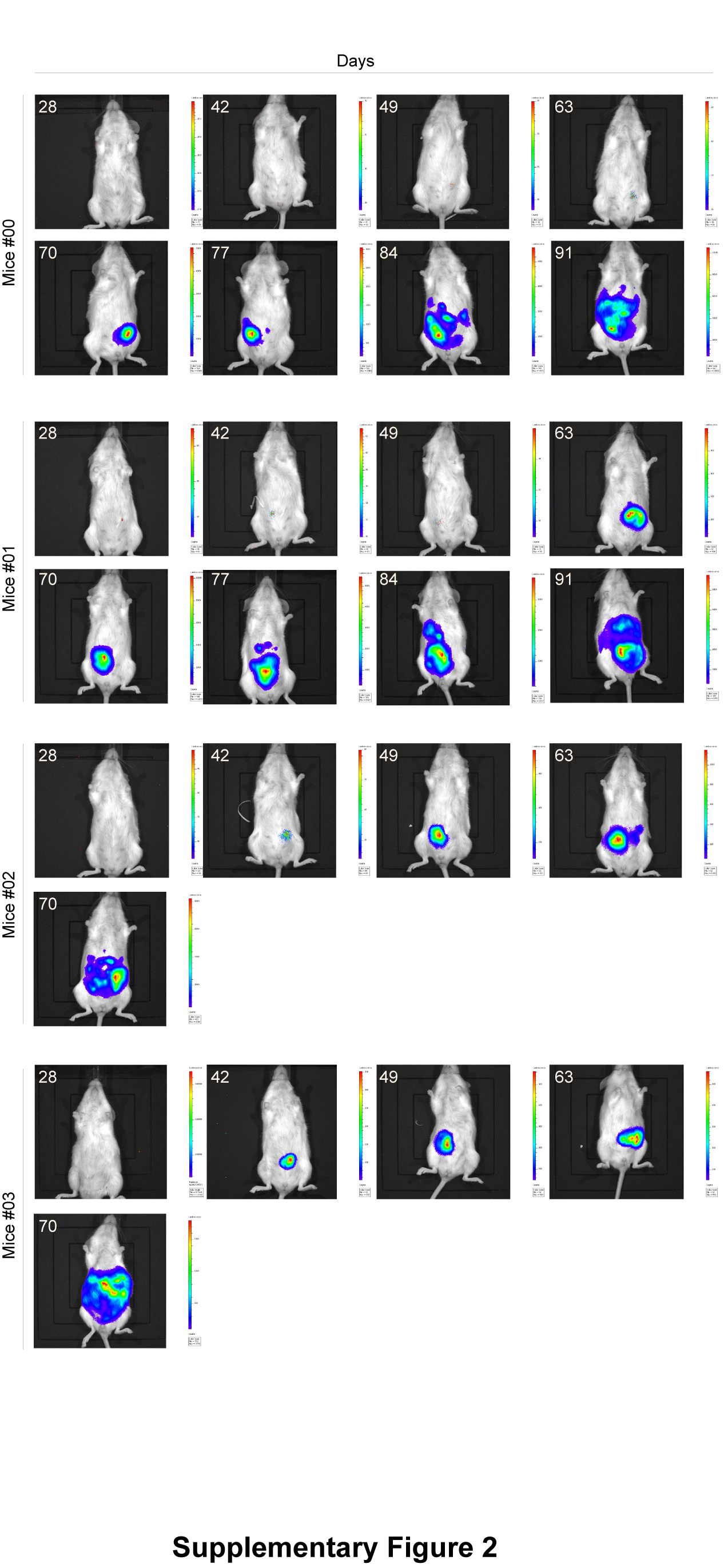

Supplement: Supplementary file 6 — Additional file 6: Figure S2. Growth and metastatization of orthotopic tumor xenografts recorded by bioimaging. Representative images of LUC-expressing PDOs orthotopically injected into the colon wall of NSG mice and monitored by bioluminescent imaging (IVIS imaging system) at different time points. [file 13046_2022_2263_MOESM6_ESM.tif]

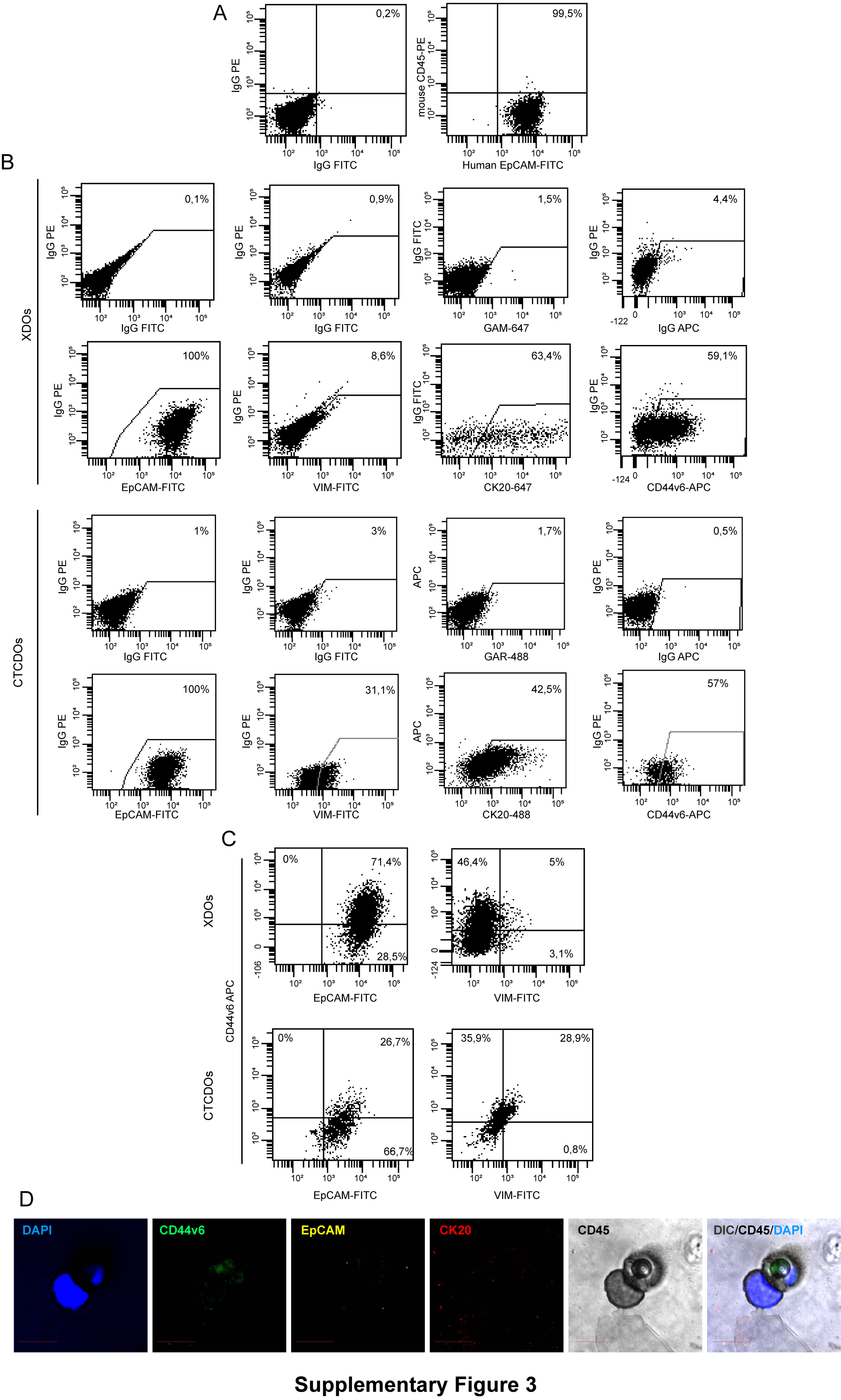

Supplement: Supplementary file 7 — Additional file 7: Figure S3. CTCDOs phenotypic characterization. (A) Flow cytometry of purified CTCDOs stained for human EpCAM and mouse CD45 expression. (B) Representative flow cytometry plots performed on XDOs and CTCDOs for the expression of: EpCAM, VIM, CK20, CD44v6 shown in Fig. 3C. (C) Representative flow cytometry plots performed on XDOs and CTCDOs for the expression of CD44v6/EpCAM and CD44v6/Vimentin Fig. 3D. (D) Representative confocal images of CD45-positive (hematopoietic) cells present on ScreenCell® filters. Cells were stained with CD45/DAB (appearing as the dark staining in the differential interference contrast/DIC brightfield), CD44v6 (green), EpCAM (yellow) and CK20 (red). Nuclei were counterstained with DAPI. Magnification 60x, 5x zoom, bar 10 μM. [file 13046_2022_2263_MOESM7_ESM.tif]

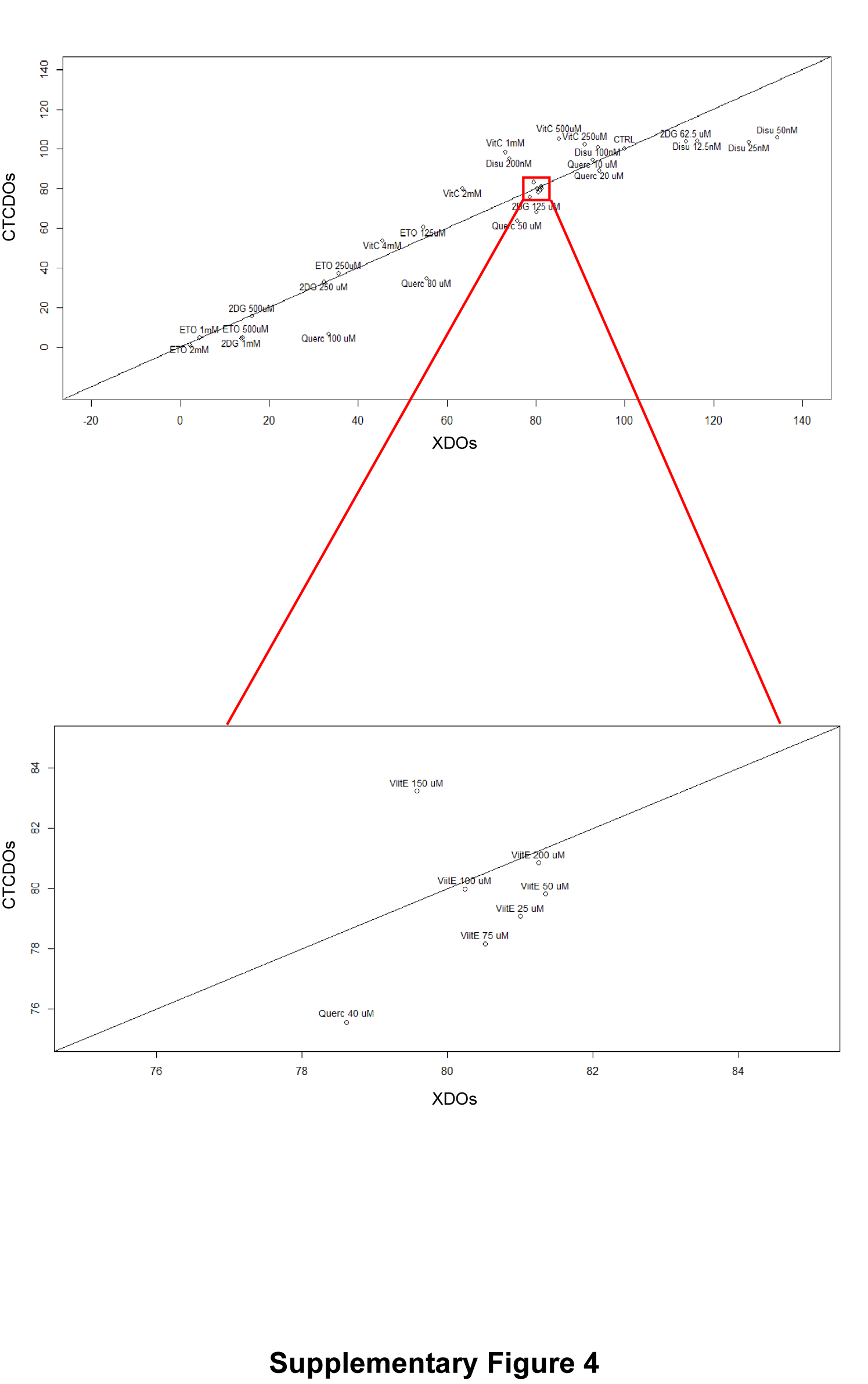

Supplement: Supplementary file 8 — Additional file 8: Figure S4. Linear regression of the effect of low toxicity compounds on CTCDOs and XDOs. Schematic representation trough linear regression of low toxicity compounds described in Fig. 5D and used at different indicated doses specified in Additional file 3: Table S2 (upper panel); detail of endpoints contained in the red square (lower panel). [file 13046_2022_2263_MOESM8_ESM.tif]

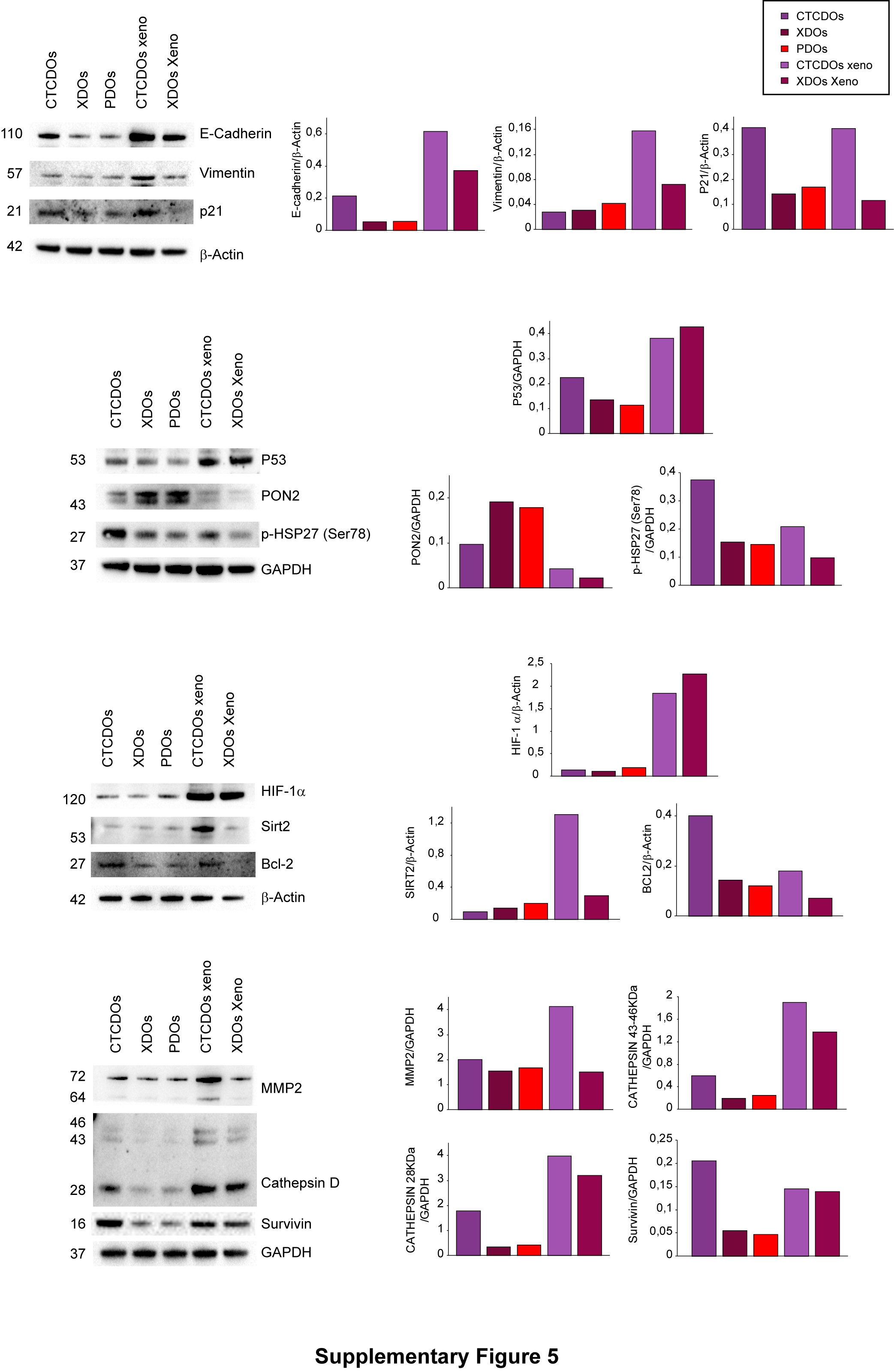

Supplement: Supplementary file 9 — Additional file 9: Figure S5. Selected protein validations on CTCDOs, XDOs, PDOs and CTCDOs/XDOs-derived xenografts. Left: immunoblot analysis of E-Cadherin, Vimentin, p21, P53, PON2, pHSP27 (Ser78), HIF-1α, Sirt2, Bcl-2, MMP2, Cathepsin D and Survivin on whole lysates of CTCDOs, XDOs, PDOs and CTCDOs/XDOs-derived xenografts (reported as CTCDOs xeno and XDOs xeno). β-actin and GAPDH were used as a loading control. Right: quantification of immunoblot experiments. [file 13046_2022_2263_MOESM9_ESM.tif]
